# An Efficient Expression and Purification Protocol for SpCas9 Nuclease and Evaluation of Different Delivery Methods of Ribonucleoprotein

**DOI:** 10.3390/ijms25031622

**Published:** 2024-01-28

**Authors:** Konstantin Evmenov, Nikolay Pustogarov, Dmitri Panteleev, Artur Safin, Elena Alkalaeva

**Affiliations:** 1 Engelhardt Institute of Molecular Biology, The Russian Academy of Sciences, 119991 Moscow, Russia; kevmenov@mail.ru (K.E.); pustogarov@gmail.com (N.P.); 2 Center for Precision Genome Editing and Genetic Technologies for Biomedicine, Engelhardt Institute of Molecular Biology, The Russian Academy of Sciences, 119991 Moscow, Russia; 3 Department of Biology, Pirogov Russian National Research Medical University, 117997 Moscow, Russia; artur-safin-96@mail.ru; 4 Institute of Higher Nervous Activity and Neurophysiology, The Russian Academy of Sciences, 117485 Moscow, Russia; mycobiota@yandex.ru

**Keywords:** SpCas9, protein purification, RNP, genome editing

## Abstract

The Clustered Regularly Interspaced Short Palindromic Repeats (CRISPR)-Cas9 system is a revolutionary tool for precise genome editing across various cell types. Ribonucleoproteins (RNPs), encompassing the Cas9 protein and guide RNA (gRNA), have emerged as a promising technique due to their increased specificity and reduced off-target effects. This method eliminates the need for plasmid DNA introduction, thereby preventing potential integration of foreign DNA into the target cell genome. Given the requirement for large quantities of highly purified protein in various Cas9 studies, we present an efficient and simple method for the preparation of recombinant *Streptococcus pyogenes* Cas9 (SpCas9) protein. This method leverages the Small Ubiquitin Like Modifier(SUMO) tag system, which includes metal-affinity chromatography followed by anion-exchange chromatography purification. Furthermore, we compare two methods of CRISPR-Cas9 system delivery into cells: transfection with plasmid DNA encoding the CRISPR-Cas9 system and RNP transfection with the Cas9-gRNA complex. We estimate the efficiency of genomic editing and protein lifespan post-transfection. Intriguingly, we found that RNP treatment of cells, even in the absence of a transfection system, is a relatively efficient method for RNP delivery into cell culture. This discovery is particularly promising as it can significantly reduce cytotoxicity, which is crucial for certain cell cultures such as induced pluripotent stem cells (iPSCs).

## 1. Introduction

Clustered Regularly Interspaced Short Palindromic Repeats-CRISPR-associated protein 9 (CRISPR-Cas9) has been a promising and accessible genome editing system since its discovery [1]. It employs a Cas9 protein, commonly isolated from *Streptococcus pyogenes* (SpCas9) [2], an endonuclease capable of inducing a double-stranded break in a DNA molecule, and a guide RNA (gRNA) that directs the protein to a specific DNA sequence [3]. This technology has been successfully applied in both in vitro and in vivo studies [4,5]. Therapies based on the CRISPR-Cas9 system are being developed for clinical use, with some already approved [6,7], with ongoing efforts towards enhancing the efficiency of this genomic editor [8,9,10]. SpCas9, a large protein consisting of 1368 amino acids with a molecular weight of 158 kDa, has a well-established domain structure and mechanism of action [2,11]. 

For SpCas9-related studies, especially its application and delivery to cells, a substantial amount of functionally active protein is required, leading to a demand for laboratory production protocols. However, due to SpCas9’s size and cell toxicity [12], obtaining this protein in sufficient quantities presents significant challenges. Numerous production methods have been described, employing different expression strains, induction conditions, and isolation and purification techniques [13,14,15,16,17]. These methods are primarily based on metal-affinity chromatography, followed by gel filtration or ion-exchange column purification. Most of the described protocols isolate SpCas9 with an additional tag, typically a His-tag, which does not appear to significantly reduce the efficiency of the genomic editor. However, for several studies, tag-free protein is required in substantial quantities.

Cas9 can be delivered into cells in three different forms: as a plasmid vector encoding both Cas9 and guide RNA (gRNA); as mRNA encoding Cas9 along with gRNA; and as ribonucleoprotein (RNP), consisting of the Cas9-gRNA complex [18]. Each form has its own advantages and potential drawbacks [19]. Delivery of the plasmid vector is relatively simple to perform [20], but it carries the risk of increased off-target effects due to the extended lifetime of the protein within the cell [21,22,23]. Additionally, there is the possibility of spontaneous integration the gene cassette into the genome of recipient cell [24], especially in case of viral vector delivery [25], which is a constraint for therapeutic protocols.

In contrast, mRNA delivery does not have these limitations as the RNA lifespan within the cell is significantly shorter and there is no risk of disrupting the genome integrity of recipient cells [26]. However, due to the lower stability of RNA, this approach is more complex and has lower efficiency of genetic editing, which is associated with intracellular degradation of RNA molecules [27,28].

The introduction of RNP appears to be the most promising for therapeutic applications, as it limits the protein lifetime in the cell, thereby reducing off-target effects [24], and maintains high editing efficiency [29,30]. Various Cas9 delivery methods, including lipofection, electroporation, cell-penetrating peptides, and microinjections, are utilized, each with varying degrees of efficacy [31]. The preferred method may vary depending on the cell type and application [32].

In this study, we present a simple and efficient method for producing functionally active SpCas9 protein. We generated a construct containing a SUMO-tag, which facilitates SpCas9 folding when expressed in bacteria [33], and evaluated its expression efficiency across different *E. coli* strains. The strain yielding the highest protein production was identified and protocols for SpCas9 production were optimized. We compared the efficiency of SpCas9 delivery into cells using different classical methods and the duration of SpCas9 presence in cells. The efficiency and kinetics of genome editing were also compared across different methods of protein delivery into cell culture.

## 2. Results

### 2.1. Optimization of SpCas9 Expression

**Figure 1:**
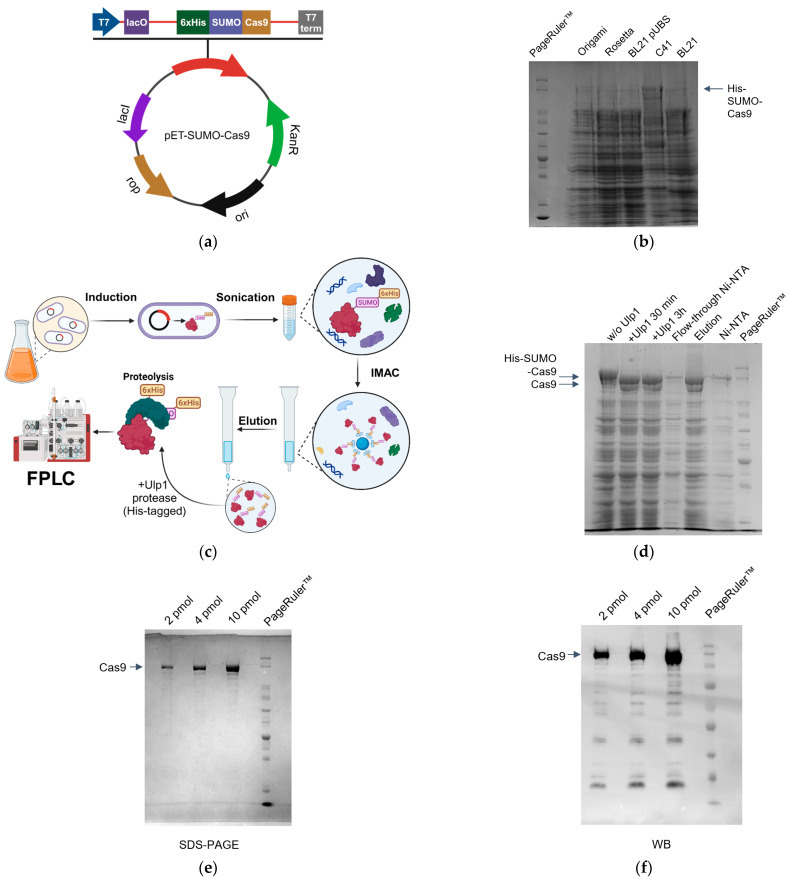
Expression and purification of SpCas9. (**a**) Scheme of the pET-SUMO-Cas9 expression vector. (**b**) SpCas9 protein expression in different *E. coli* DE3 strains; 10% SDS-PAGE of various strains lysates after induction. (**c**) Schematic representation of protein purification process. (**d**) Purification of SpCas9; 10% SDS-PAGE after partial proteolysis and binding with Ni-NTA resin. (**e**) 10% SDS-PAGE of purified protein. (**f**) Western blot analysis with antibody against the C-terminal region of SpCas9.

In the field of molecular biology, *Escherichia coli* (*E. coli*) bacteria serve as a highly adaptable platform for the production of recombinant proteins, a fact underscored by extensive research and utilization across various studies [34,35]. Various strains of *E. coli* have been engineered and employed, reflecting the unique molecular properties of the resulting proteins, including their structure, activity, and size [36]. The production of SpCas9is significantly difficult due to the protein’s large size and potential cytotoxicity towards the host *E. coli* strains. Additionally, the *SpCas9* gene in expression constructs commonly is encoding by eukaryotic codons which complicates its expression. To address these challenges, we developed a pET-SUMO-Cas9 expression cassette. This construct was built upon the pET-SUMO vector (K30001 Invitrogen, Waltham, MA, USA), incorporating a SUMO sequence that increases the solubility of the expressed protein and facilitates its correct folding [37] (Figure 1a). This cassette also includes a sequence that allows for the cleavage of all additional fragments using the Ulp1 protease. Consequently, the target protein’s sequence does not contain any additional amino acids, ensuring a perfect match with the native protein.

The pET-SUMO-Cas9 was expressed in several widely distributed *E. coli* strains, including BL21, BL21 with helper plasmid pUBS, Rosetta, Origami, and C41 (Table 1).

SpCas9 demonstrated highest expression in the C41 strain of *E. coli* (Figure 1b). as compared with the expression levels of SpCas9 in the strains BL21, Origami, Rosetta, and pUBS. Thus, further purification proceeded with the use of the C41 strain. In addition to the selection of strain, the expression conditions of SpCas9 were also optimized. This involved testing a range of conditions, including different concentrations of IPTG used for induction (from 0.1 to 1 mM), bacterial cell cultivation temperatures (from 16 °C to 37 °C), and durations of cultivation (from 2 to 24 h). Finally, it was determined that the optimal induction conditions were a concentration of 0.5 mM IPTG and a temperature of 18 °C for overnight cultivation. These conditions allowed the efficient expression of SpCas9 in the C41 strain, demonstrating the effectiveness of this approach in producing this recombinant protein.

### 2.2. SpCas9 Purification Protocol

For the subsequent production of Cas9 using the C41 strain, a 250 mL cell suspension was utilized. The initial step of protein purification from the cell lysate involved affinity chromatography on Ni-NTA agarose (Figure 1c). Following elution from Ni-NTA agarose, the SpCas9 preparation was found to contain a substantial amount of impurities (Figure 1d, lane 1). Subsequent to this, SUMO-tag cleavage was proceeded using Ulp1 protease. One hour of incubation in the presence of the protease was sufficient for complete cleavage of the SUMO sequence (Figure 1d, lanes 2 and 3).

In the second phase of purification, post proteolysis of Ulp1, SpCas9 bound to Ni-NTA agarose during the removal of the His-SUMO-tag. This resulted in a significant reduction in the yield of the target protein (Figure 1d, lane 4). Interestingly, previously described methods for protein isolation with a partial proteolysis step did not exhibit loss of this protein during the re-incubation step with Ni-NTA [38].

For further purification, ion-exchange chromatography was conducted on two HiTrapQ and HiTrapSP columns (Figure S1B). This yielded a protein preparation with a purity of approximately 95%, lacking any additional tag at the N-terminus, as shown in Figure 1e,f and Figure S1B. A Western blot of the obtained preparation using specific antibodies to Cas9 revealed the presence of partially degraded forms of the Cas9 protein, even though a protease inhibitor was used throughout all stages of isolation. These additional bands were also visible in other protocols of SpCas9 purification [38]; thus, despite the amounts of degraded forms being low, this issue is thought to be common and warrants consideration when conducting studies and estimating the concentration of the active form of the protein. The general scheme of Cas9 expression and purification is depicted in Figure 1c.

The protein yield was estimated by calculating the amount of the obtained protein and accounting for the volume of the culture medium used. From 250 mL culture medium, we purified 0.97 mg of SpCas9. Therefore, the final yield of SpCas9 was 4 mg from 1 L of culture, which is commendable for such large proteins. The abundant amount of SpCas9 obtained allows for additional purification by various methods without the risk of losing a significant proportion of the material. Analysis of the purified SpCas9 preparation for RNases did not reveal their presence, as shown in Figure S2. Therefore, we have established a simple and efficient method for the preparation of SpCas9 in a bacterial expression system.

### 2.3. RNP Assembly and Evaluation of Its Activity In Vitro

Subsequently, the Ribonucleoprotein (RNP) complexes were formed based on the obtained recombinant SpCas9. This was achieved by obtaining a gRNA with a spacer targeting the Green Fluorescent Protein (GFP) sequence (Table S1). An RNP complex was assembled using this gRNA by incubating SpCas9 with the gRNA.

**Figure 2:**
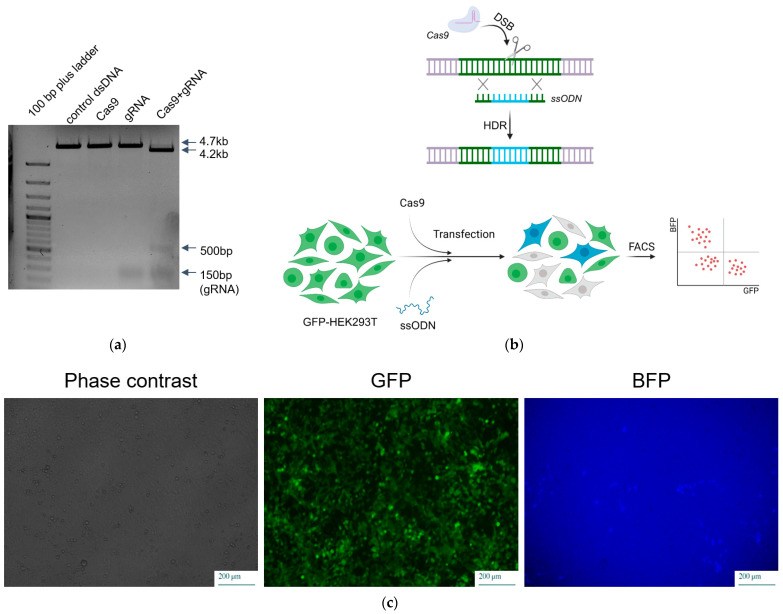
Ribonucleoprotein functional activity. (**a**) In vitro analysis of RNP complex activity. (**b**) Graphical representation of green fluorescent protein (GFP) conversion to blue fluorescent protein (BFP). (**c**) Fluorescent microscopy of HEK293T cell line after the delivery of RNP complex with ssODN.

To assess its nuclease activity, the RNPs were incubated with the pre-linearized plasmid pEGFP-N1 (Table S1). The functional activity of recombinant SpCas9 was confirmed through plasmid cleavage (Figure 2a).

### 2.4. Comparison of Delivery Methods of SpCas9 into Mammalian Cells

**Figure 3:**
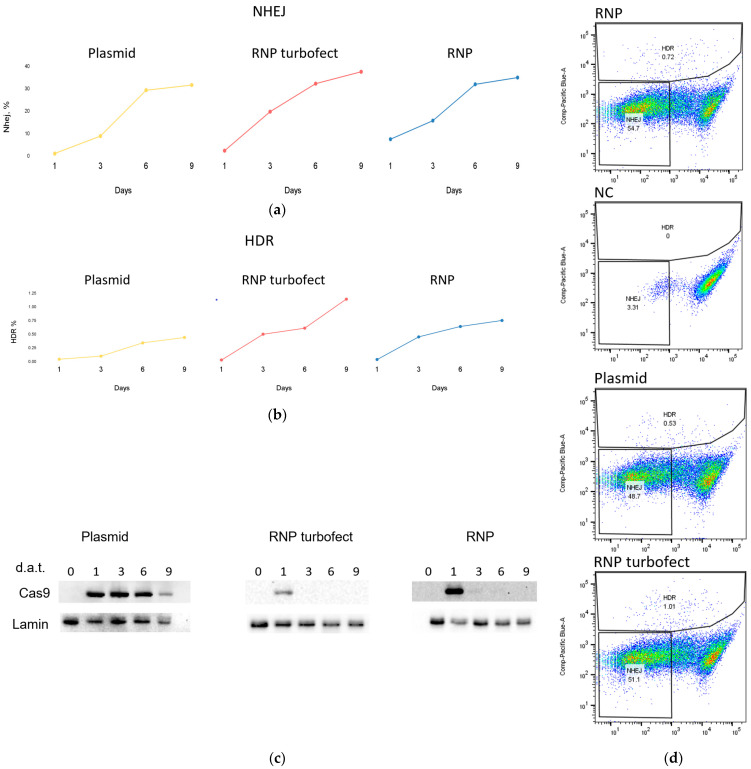
Analysis of different CRISPR-Cas9 delivery methods into HEK293T. Plasmid transfection and RNP transfection via TurboFect and RNP self-delivery were evaluated at 1, 3, 6, 9 days after transfection. (**a**) Non-homologous end joining (NHEJ). (**b**) Homology-directed repair (HDR). (**c**) Western blot analysis with antibodies against SpCas9 (d.a.t.—days after transfection). (**d**) Flow cytometry plots for GFP-to-BFP reporter cells showing frequency of HDR (BFP+) and NHEJ (BFP−/GFP−). Cells were transfected with plasmid (plasmid transfection), RNP (RNP transfection) or exposed to RNP itself (RNP) A negative control (NC) included. Representative density plots are shown at day 9 post transfection.

To examine the activity of the obtained RNP complex ex vivo, we compared its activity with the plasmid delivery method of SpCas9. For this analysis, HEK293 cells were transfected with both the RNP complex (SpCas9+gRNA) and plasmid px458 (#48138 Addgene, Watertown, MA, USA) encoding the Cas9 protein with a gRNA directed to the GFP sequence. We also evaluated the activity of the CRISPR-Cas9 genome editing system using a previously described approach based on editing the *GFP* gene inserted into the genome of a cell line [39]. This method involves editing the *GFP* gene in the presence of donor DNA carrying a mutation, leading to either a gene frame disruption at non-homologous end joining (NHEJ) or a mutation that changes the green fluorescence spectrum to blue via homologous recombination (HDR) (Figure 2b,c). The formation of blue and colorless cells was detected using flow cytometry (Figure 3d). This allowed us to determine the efficiency of NHEJ and HDR at 1, 3, 6 and 9 days after transfection (Figure 3a,b).

Simultaneously, we assessed the protein lifetime in cells after transfection with plasmid or RNP complex using Western blotting at 1, 3, 6 and 9 days after transfection (Figure 3c). As expected for transfection with a plasmid encoding Cas9, we detected comparable protein levels on days 1, 3, and 6 in cell culture. However, when transfected with the RNP complex, the protein was detectable on the first day and undetectable on Western blot as early as day 3. These dynamics are generally similar to those described previously in the literature [24].

The NHEJ level was higher with RNP and reached almost maximum values on day 6. HDR level increased on all 9 days and was higher in the presence of RNP than in the presence of plasmid. This demonstrated that the resulting recombinant RNP edits the genome more efficiently than SpCas9 expressed directly in cells. At the same time, its lifetime is limited to one or two days, which reduces the possibility of off-targets.

As a control for RNP transfection, we used an RNP complex without any transfection reagent. We were surprised to detect a signal from Western blotting after incubation of cells with RNP complex without transfection reagent. Its dynamics was similar to that during transfection with RNP complex—it appeared on the first day after transfection and was practically undetectable on the third day. Although we initially assumed that this was merely an artifact, in an additional experiment using different transfection reagents, we obtained a similar result (Figure S3). RNPs without transfection reagent and RNPs in the presence of lipofectamine CRISPRMAX and TurboFect were detectable in cells on day 1 and day 2. Furthermore, when we evaluated the levels of NHEJ and HDR, which we also performed on days 3, 6, and 9, we obtained comparable values of the investigated parameters between RNP genome editing upon transfection with TurboFect and control RNP, indicating not only the spontaneous internalization of the RNP complex through the membrane of HEK293T cells but also the preservation of its functional properties.

## 3. Discussion

In this study, we introduce a method for the expression and purification of the SpCas9 nuclease as a fusion protein with a SUMO-tag. Despite the existence of several variations of SpCas9 isolation strategy in the literature [13,14,15,16,17,23], the vast number of studies utilizing SpCas9 requires the development of new reproducible protocols for obtaining functionally active protein. A key advantage and novelty of our approach is its high productivity and simplicity, which allowed us to obtain an extremely large amount of SpCas9 in bacterial cells. An additional advantage of this method is the absence of any tags in the final preparation of SpCas9, which can potentially increase its activity and be useful for structural, interaction, biophysical, and chemical modification studies [40,41].

The large size of SpCas9 reduces the efficiency of its production by bacterial cells, due to the unique characteristics of protein folding in bacterial cells compared to eukaryotic expression systems [42]. Another challenge lies in the functional properties of SpCas9. As a programmable endonuclease, its overexpression leads to cytotoxic effects in cells, thereby reducing protein production levels [42]. Furthermore, the presence of rare codons in the Cas9 sequence negatively impacts the final product yield [43]. These issues contribute to the ongoing search for an optimal protocol to produce recombinant Cas9 protein [17,44]. To address these challenges, we chose to express the protein as a fusion variant with a SUMO-tag, which increases the solubility of the bound proteins. To minimize the cytotoxicity of the protein, we selected an *E. coli* strain with the least sensitivity to this effect. As a result, strain C41 was selected, which possesses mutations that slow down cell growth and thereby increase tolerance to protein toxicity.

Following the first round of purification on Ni-NTA agarose, we initially planned to repeat the chromatography on Ni-NTA agarose to remove the detached His-SUMO-Tag and His-Ulp1 from the sample. However, we discovered that under the conditions we used, SpCas9 bound to the resin, which significantly reduced the quantity of the final product. Consequently, we decided to skip this step and used an anion-exchange column in this protocol instead. However, it should be noted that this protocol can potentially be modified to minimize the binding of SpCas9 to the resin, for instance, by increasing the concentration of imidazole during incubation of the cell lysate with the resin or by adjusting its concentration in the elution buffer. While this would require further research to optimize the protocol, such an approach may be suitable for less-equipped laboratories, while maintaining the total protein yield and slightly reducing the purity of the preparation. Following all purification steps, we obtained approximately four milligrams of protein from one liter of cell suspension with a purity exceeding 95%, surpassing values obtained using other protocols [13,17].

Next, we tested the functional activity of recombinant SpCas9 and evaluated the dynamics of NHEJ and HDR. RNP, containing Cas9, could be delivered into cells using various methods: microinjection [45], electroporation [24,46], cell-penetrating peptides [47,48], DNA nanoclew [49], gold nanoparticles [50], iTOP [51], and others. However, RNP transfection remains the simplest and cheapest method, which, as we observed, is not always effective. For the transfection of the RNP complex, we used TurboFect, which, while not a specific reagent for RNP transfection, has been reported to provide an acceptable level of transfection and is a common transfection reagent alongside lipofectamine [52]. In a more detailed study of RNP dynamics in cells, we used lipofectamine CRISPRMAX, which resulted in significantly higher levels of transfection (Figure S3). The most surprising part of our findings was the spontaneous delivery of RNP into HEK293T cells. The delivery of RNP into cells was confirmed by Western blotting and flow cytometry, which detected cells subjected to genome editing.

How can Cas9 RNP be internalized into cells in the absence of transfection reagents? We suppose that the significant positive charge of SpCas9 (+22) could play an important role in such delivery. The positive charge of SpCas9 makes it similar to polycationic proteins [53] and penetrating peptides [47,48], in which positively charged residues facilitate interaction with negatively charged cell surface proteoglycans, ensuring cellular uptake. These positively charged proteins access the interior of cells during endocytosis. Further studies are needed to determine the conditions of spontaneous delivery and to explore the specific features of the cell lineage that cause such delivery. Nevertheless, the search for new ways to deliver RNPs with reduced cytotoxicity and immunogenicity is a promising avenue for various fields, from therapeutic practice to work with induced pluripotent stem cells (iPSCs).

## 4. Materials and Methods

### 4.1. Plasmids Construction

The SpCas9 expression plasmid pET-SUMO-Cas9 with 3x-FLAG and SV40-NLS at the N-terminus and a nucleoplasmin-NLS at the C-terminus was amplified via PCR using Q5 polymerase (NEB, Ipswich, MA, USA), the plasmid px458 (Addgene#48138), and the primers petSUMO_3xFLAG_F and petSUMO_CAS9_ver1_R. The petSUMO vector sequence was amplified from the petSUMO plasmid (K30001 Invitrogen, Waltham, MA, USA) using Q5 polymerase (NEB, Ipswich, MA, USA) and primers petSUMO_F and petSUMO_R. The pET-SUMO-Cas9 plasmid was obtained with the Gibson Assembly^®^ Master Mix (NEB, Ipswich, MA, USA) according to the manufacturer’s protocol. The resulting plasmid included the sequences of 6xHis-tag necessary for protein purification and the SUMO-tag sequence.

Modified px458 plasmid was used for the expression of SpCas9 and genome editing analysis in HEK239T cells. Initially, the EGFP gene was removed from the px458 vector by restriction by *EcoRI* sites. A spacer sequence targeting the GFP gene was introduced into the plasmid sequence using primers aGFPsp2F and aGFPsp2R. The primers were treated with T4 PNK kinase (NEB, Ipswich, MA, USA) followed by duplex formation at a temperature gradient from 95 °C to 25 °C over a 15 min period. The primer duplex was cloned into the vector by Golden Gate Cloning using BbsI restriction enzyme (NEB, Ipswich, MA, USA) and T4 DNA Ligase (Promega, Madison, WI, USA). Sequences of all oligos are listed in Table S1.

### 4.2. Expression and Purification of SpCas9 Protein

Origami B, Rosetta, BL21(pUBS), C41, and BL21 strains of DE3 cells were transformed with the pET-SUMO-Cas9. After that, the cells were transferred into liquid LB with a doubled concentration of peptone and yeast extract. After reaching OD600 0.4 o.u. at 37 °C, cells were induced with 0.1, 0.25, 0.5, 0.75 or 1 mM IPTG and incubated for 2, 4, 8, 16 or 24 h at 16 °C, 20 °C, 25 °C or 37 °C. The protein concentration was estimated using SDS-PAGE.

The precipitate obtained from the C41(DE3) cells (250 mL) expressing 6xHis-SUMO-Cas9 was resuspended in 25 mL of lysis buffer, containing 20 mM Tris-HCl (pH 7.5), 500 mM KCl, 10% glycerol, 1 mM PMSF, 1 mM DTT, and disintegrated using ultrasound. The lysate underwent centrifugation at 20,000× *g* at 4 °C for 30 min. The supernatant was incubated with 200 μL of 50% Ni-NTA agarose for 2 h at 4 °C. The resin was subsequently washed and the proteins were eluted. Then, the proteins were dialyzed against the cleavage buffer and subjected to proteolytic cleavage using 6xHis-labeled Ulp1 protease. The proteins were further dialyzed against buffer containing 20 mM HEPES (pH 7.5), 100 mM KCl, 10% glycerol, and 1 mM DTT and purified using ion-exchange chromatography with a HiTrapSP column (Cytiva, USA). The final product was dialyzed against a storage buffer, frozen in liquid nitrogen, and stored at −70 °C.

### 4.3. gRNA Preparation

The guide RNA was synthesized using the EnGen sgRNA Synthesis Kit, S. pyogenes (NEB, Ipswich, MA, USA), according to the manufacturer’s protocol. aGFPOligCas oligo (Evrogen, Moscow, Russia) contained the sequence of the T7 promoter, the spacer sequence against the GFP gene, and the homologous region required for in vitro synthesis of the DNA template.

Obtained samples were treated with DNase and purified via phenol-chloroform extraction (pH 5.0). The samples were precipitated with ethanol and dissolved in ddH2O. The RNA was further purified using Amersham MicroSpin G-50 Columns gel-filtration columns (Cytiva, Chicago, IL, USA).

### 4.4. RNP Complexes Preparation

RNP complexes were synthesized according to the protocol described in “In vitro digestion of DNA with Cas9 Nuclease, S. pyogenes” (NEB, Ipswich, MA, USA). Equimolar amounts of gRNA and SpCas9 were mixed to a final concentration of 300 nM in 30 μL of NEBuffer 3.1 (NEB, Ipswich, MA, USA). The mixture was gently mixed and incubated at room temperature for 10 min.

The activity of the RNP complex in vitro was further demonstrated by incubating the complex with the pEGFP-N1 plasmid linearized by the *NotI* restriction site (Clontech, Changhai, China) for 30 min. The mixture was then separated using electrophoresis in 1% agarose gel and DNA fragments were visualized with ethidium bromide. Activity was determined by the presence of DNA fragments with initial lengths of 4700 bp and cleaved forms of 4200 bp and 500 bp.

### 4.5. Mammalian Cell Line and Transfection

The HEK293 eukaryotic cell line was cultured in a DMEM culture medium (PanEco, Moscow, Russia) supplemented with 10% fetal bovine serum (Dia-M, Moscow, Russia) and maintained in 5% CO_2_ and a 98% humidity atmosphere. The transfection of the HEK293 cells with plasmid and RNP was performed using Lipofectamine™ 3000 Transfection Reagent (Invitrogen, USA), Lipofectamine™ CRISPRMAX (Invitrogen, Waltham, MA, USA) and TurboFect (ThermoFisher, Waltham, MA, USA) in accordance with the manufacturer’s instructions. For plasmid transfection, 300 ng of Cas9 coding vector and 200 ng of ssODN (see Table S1) were used. For the RNP transfection, 20 pmol (3300 ng) of the complex was used; 200 ng of ssODN oligonucleotide was used for cotransfection to achieve HDR.

The HEK293T stable GFP transfected cell line was obtained using transfection with pEGFP-N1 plasmid vector (#V012021 Clontech, Changhai, China) and subsequently subcloned. The level of GFP expression was visually examined until a stably transfected cell line was obtained.

### 4.6. Flow Cytometry

Flow cytometry was performed using a BD LSRFortessa™ Cell Analyzer. HEK293T cells seeded in a 24-well plate were pelleted using centrifugation at 1000× *g* for 1 min and resuspended in 500 μL of FACS buffer solution containing 1% BSA in PBS buffer. A total of 40,000–50,000 cells were analyzed for GFP−/BFP− cells (end-joining pathways) and BFP+ cells (HDR pathway) 3, 6, and 9 days after transfection. Untreated cells were used as a negative control. The data were subsequently analyzed using FlowJo_v10.8.1 software (Ashland, OR, USA).

### 4.7. Western Blot Analysis

The presence of SpCas9 protein in the eukaryotic cell line was analyzed using Western blotting. HEK293 cells were transfected with expression vector, based on the px458 vector, or with RNP complexes. Post-transfection, cells were incubated for 1, 3, 6, or 9 days, resuspended, pelleted, and lysed in Laemmli buffer containing 50 mM Tris-HCl (pH 6.8), 100 mM b-MeEtOH, 50 mM DTT, 1% SDS, 0.01% Bromphenol blue, and 10% glycerol. The lysates were separated using electrophoresis in 10% SDS-PAGE in glycine buffer. The membrane was stained with Anti-CRISPR/Cas9 (Sigma-Aldrich, Burlington, MA, USA) or Anti-His (Sigma-Aldrich, Burlington, MA, USA) antibodies. The number of lysed cells was normalized to the expression level of the housekeeping gene Lamin (Thermo Fisher, Waltham, MA, USA).

## Figures and Tables

**Table 1:** *E. coli* strains used in the present study.

Stain	Source	Features
Rosetta	#70954 Novagen, Darmstadt, Germany	Expression of eukaryotic proteins containing codons rarely used in *E. coli* (AGG, AGA, AUA, CUA, CCC, GGA) is enhanced.
Origami B	#70836 Novagen, Darmstadt, Germany	Efficiently expresses and folds disulfide bond-containing proteins.
BL21	#200133 Stratagene, La Jolla, CA, USA	The regular BL21 (DE3) strain contains the lambda DE3 prophage that carries the gene for T7 RNA polymerase under control of a lacUV5 promoter, allowing expression of the T7 RNA polymerase to be induced with IPTG.
BL21-pUBS	Lab collection	Plasmid pUBS-520 was introduced, supplying recombinant bacteria with high levels of rare tRNA-Arg.
C41	#CMC0017 Sigma-Aldrich, Burlington, MA, USA	Expression of toxic proteins.

## Data Availability

The data that support the findings of this study are contained within the article and the supporting information. All source data generated for this study are available from the corresponding author (Elena Alkalaeva; alkalaeva@eimb.ru) upon reasonable request.

## Supplementary Materials

The following supporting information can be downloaded at: https://www.mdpi.com/article/10.3390/ijms25031622/s1.
