# Supplementary material for: An Efficient Expression and Purification Protocol for SpCas9 Nuclease and Evaluation of Different Delivery Methods of Ribonucleoprotein
Source: Int J Mol Sci. 2024 Jan 28;25(3):1622. doi: 10.3390/ijms25031622 (PMC10855156; doi:10.3390/ijms25031622)
Supplement: Supplementary file 1 [file ijms-25-01622-s001.zip › ijms-2827034-supplementary.pdf]

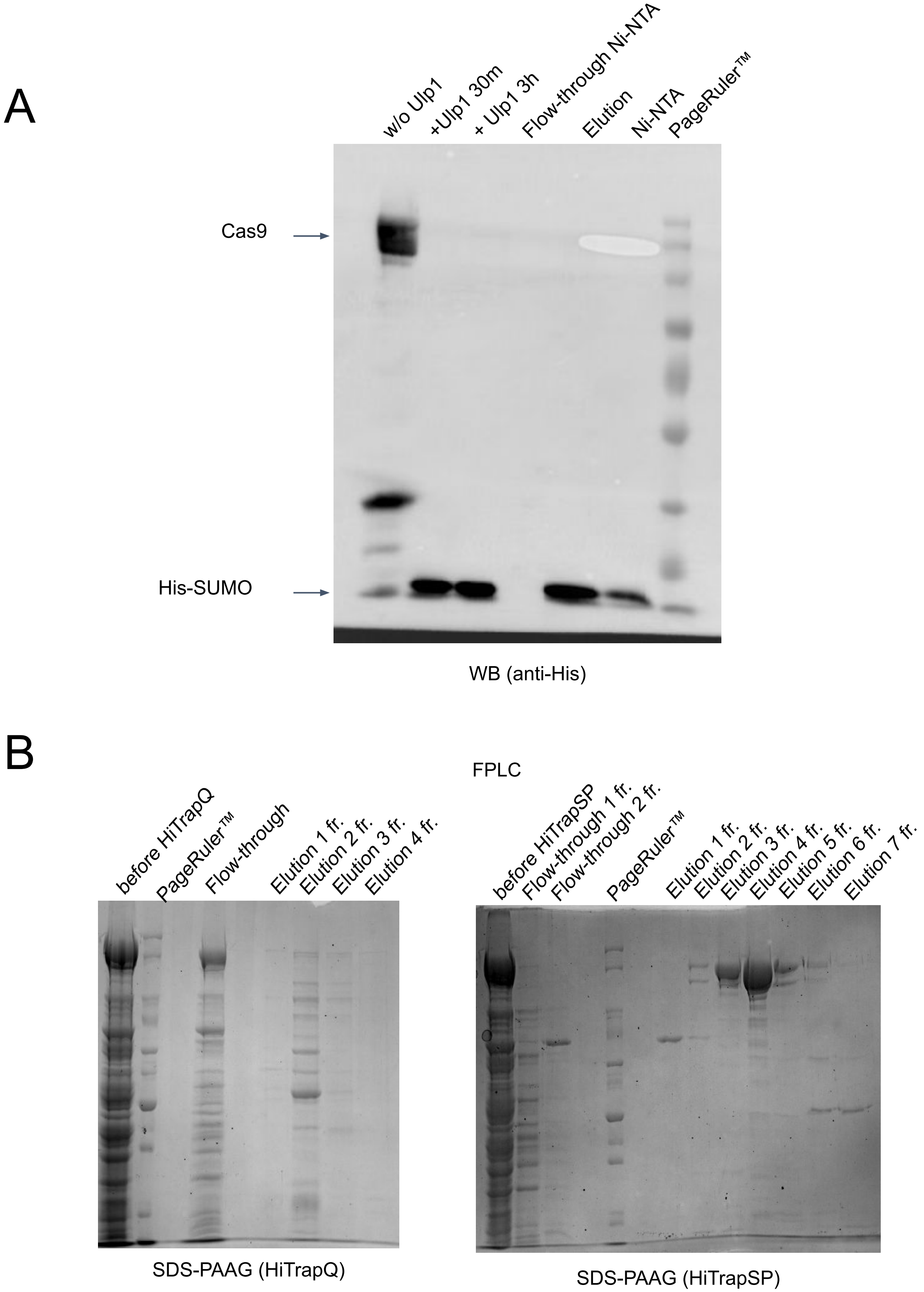

Figure S1. Stages of SpCas9 purification. (A) Western blot analysis of partial hydrolysis stages by Ulp1 protease, with antibody staining against the His-tag. (B) SDS-PAGE analysis of fractions obtained via ion exchange chromatography.

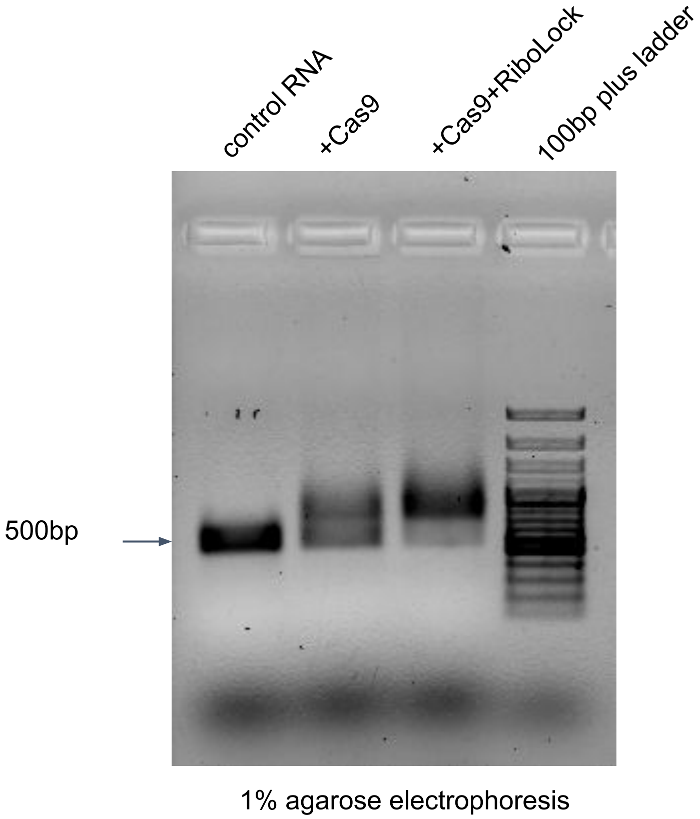

Figure S2. Examination of RNase Contaminant Presence in the Obtained Cas9 Protein Preparation.

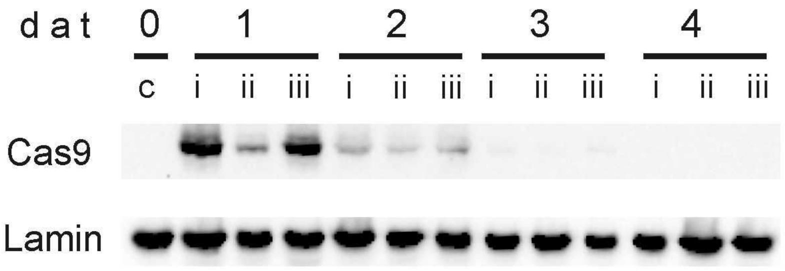

i - RNP
ii - RNP+ TurboFect
iii - RNP+ Lipofectamine CRISPRMAX

Figure S3. Western Blot Analysis of SpCas9 in HEK293T cell line post RNP complex delivery using TurboFect, lipofectamine CRISPRMAX, and RNP itself. Samples were collected on days 1, 2, 3, and 4.

Table S1. Oligos used in this study

petSUMO_3xFLAG_F	5’-GAGAACAGATTGGTGGTATGGACTATAAGGAC CACGAC
petSUMO_CAS9_ver1_R	5’-CCGAATAAATACCTAAGCTCAGCCCTCTCCACT GCC
petSUMO_F	5' -AGCTTAGGTATTTATTCGGCGCAAAGTG
petSUMO_R	5' -ACCACCAATCTGTTCTCTGTGAGC
aGFPOligCas	5'-TTCTAATACGACTCACTATAGGCTCGTGACCAC CCTGACCTAGTTTTAGAGCTAGA
aGFPsp2F	5' -CACCGCTCGTGACCACCCTGACCTA
aGFPsp2R	5' -AAACTAGGTCAGGGTGGTCACGAGG
SSODN	5' -GGC ATG GCG GAC TTG AAG AAG TCG TGC TGC TTC ATG TGG TCG GGG TAG CGG CTG AAG CAC TGC ACC CCG TGG CTC AGG GTG GTC ACG AGG GTG GGC CAG GGC ACG GGC AGC TTG CCG GTG GTG CAG ATG AAC TTC AGG GT
